# A New High Throughput Sequencing Assay for Characterizing the Diversity of Natural *Vibrio* Communities and Its Application to a Pacific Oyster Mortality Event

**DOI:** 10.3389/fmicb.2019.02907

**Published:** 2019-12-20

**Authors:** William L. King, Nachshon Siboni, Tim Kahlke, Timothy J. Green, Maurizio Labbate, Justin R. Seymour

**Affiliations:** ^1^School of Life Sciences, University of Technology Sydney, Ultimo, NSW, Australia; ^2^Climate Change Cluster, University of Technology Sydney, Ultimo, NSW, Australia; ^3^Centre for Shellfish Research, Vancouver Island University, Nanaimo, BC, Canada

**Keywords:** *Vibrio*, *Vibrio* communities, seawater, oyster (*Crassostrea gigas*), DNA sequencing, marine microbiology, *hsp60*

## Abstract

The *Vibrio* genus is notable for including several pathogens of marine animals and humans, yet characterization of *Vibrio* diversity using routine 16S rRNA sequencing methods is often constrained by poor resolution beyond the genus level. Here, a new high throughput sequencing approach targeting the heat shock protein (*hsp60*) as a phylogenetic marker was developed to more precisely discriminate members of the *Vibrio* genus in environmental samples. The utility of this new assay was tested using mock communities constructed from known dilutions of *Vibrio* isolates. Relative to standard and *Vibrio*-specific 16S rRNA sequencing assays, the *hsp60* assay delivered high levels of fidelity with the mock community composition at the species level, including discrimination of species within the *Vibrio harveyi* clade. This assay was subsequently applied to characterize *Vibrio* community composition in seawater and delivered substantially improved taxonomic resolution of *Vibrio* species compared to 16S rRNA analysis. Finally, this assay was applied to examine patterns in the *Vibrio* community within oysters during a Pacific oyster mortality event. In these oysters, the *hsp60* assay identified species-level *Vibrio* community shifts prior to disease onset, pinpointing *V. harveyi* as a putative pathogen. Given that shifts in the *Vibrio* community can precede, cause, and follow disease onset in numerous marine organisms, there is a need for an accurate high throughput assay for defining *Vibrio* community composition in natural samples. This *Vibrio*-centric *hsp60* sequencing assay offers the potential for precise high throughput characterization of *Vibrio* diversity, providing an enhanced platform for dissecting *Vibrio* dynamics in the environment.

## Introduction

The *Vibrio* genus is a group of Gram-negative marine bacteria that are ubiquitous in a number of different aquatic environments, including estuaries, the open ocean, and the deep-sea (Simidu and Tsukamoto, 1985; Thompson et al., 2004; Siboni et al., 2016). The *Vibrio* genus comprises high levels of metabolic diversity and can play important ecological roles in marine biogeochemical cycling (Urdaci et al., 1988; Svitil et al., 1997; Chimetto et al., 2008; Hunt et al., 2008; Grimes et al., 2009), but are most recognized for their often ecologically important relationships with a wide range of aquatic organisms including bivalves, cephalopods, polychaetes, fish, corals, and algae (Lee and Ruby, 1994; Grisez et al., 1997; Hood and Winter, 1997; Raguenes et al., 1997; Ben-Haim and Rosenberg, 2002; Nyholm and Nishiguchi, 2008; Miyashiro and Ruby, 2012; Lemire et al., 2015; Tout et al., 2015). Notably, while some of these relationships include mutualistic interactions (Huq et al., 1983; Thompson et al., 2006), many are pathogenic with diverse *Vibrio* species causing disease in aquatic animals (Goarant et al., 2000; Kushmaro et al., 2001; Ben-Haim and Rosenberg, 2002; Becker et al., 2004; Frans et al., 2011; Geng et al., 2014; Vezzulli et al., 2015) and within aquaculture settings, sometimes resulting in significant economic losses (Lafferty et al., 2015). Notably, some *Vibrio* species are also dangerous human pathogens (Daniels and Shafaie, 2000).

Given their ecological, economic, and human health significance, assessing *Vibrio* diversity and the presence of specific *Vibrio* species in the environment is an important objective and a wide suite of both culture-dependent (Donovan and Van Netten, 1995; Grisez et al., 1997) and -independent techniques have been applied (Lane et al., 1985; Dorsch et al., 1992). Among culture-independent techniques, 16S rRNA sequencing has been widely used to characterize *Vibrio* community diversity and composition, but typically delivers poor species-level resolution, particularly among highly genetically related *Vibrio* species (Nagpal et al., 1998; Gomez-Gil et al., 2004; Pascual et al., 2010; Cano-Gomez et al., 2011), thereby limiting examination of intra-genus heterogeneity (Poretsky et al., 2014). Previous attempts to employ *Vibrio*-specific 16S rRNA primers have incrementally improved the taxonomic resolution when characterizing *Vibrio* diversity (Yong et al., 2006) but the proportion of sequences that can be unambiguously assigned to *Vibrio* species often remains low (Siboni et al., 2016). Due to the inherent inadequacies of the 16S rRNA gene for correctly identifying *Vibrio* species, another gene target encoding the heat shock protein 60 (*hsp60*, also known as *groEL* or *cpn60*), a type I chaperonin protein that assists in protein folding (Hemmingsen et al., 1988; Farr et al., 2000; Brinker et al., 2001), has been proposed as a good candidate for *Vibrio* phylogenetic studies (Kwok et al., 2002). Previous studies utilizing *hsp60* have primarily relied on *Vibrio* isolates for species identification (Preheim et al., 2011; Szabo et al., 2012; Silvester et al., 2017), but a recent study applied universal *hsp60* primers to characterize *Vibrio* community diversity using high throughput amplicon sequencing (Jesser and Noble, 2018). While this approach delivered improved *Vibrio* species identification relative to 16S rRNA, it used universal, rather than *Vibrio*-centric, *hsp60* primers and required bioinformatic filtering for *Vibrio* assigned species, as is often required for 16S rRNA sequencing.

One of the many areas where the development of a high precision assay for determining *Vibrio* community diversity would have great utility is within the aquaculture industry, where *Vibrio* infections cause substantial losses in stock and profits (Gay et al., 2004; Lafferty et al., 2015; Lemire et al., 2015; Bruto et al., 2017; Green et al., 2019), but the precise identity of the pathogen is often not well resolved or incorrectly assigned to the wrong species (Sawabe et al., 2013; Richards et al., 2014; Dubert et al., 2017; Green et al., 2019). Some *Vibrio* species, including *Vibrio splendidus* and *Vibrio coralliilyticus*, have negative impacts on oyster cultivation by causing mortality in hatcheries (Sugumar et al., 1998; Takahashi et al., 2000; Elston et al., 2008; Richards et al., 2015; King et al., 2019a). Outside of hatchery settings, a number of *Vibrio* species have been identified as oyster pathogens (Waechter et al., 2002; Saulnier et al., 2010; Duperthuy et al., 2011; Wendling et al., 2014; Bruto et al., 2017; Go et al., 2017; King et al., 2019a). Because of the complexity of oyster diseases, direct evidence for the involvement of *Vibrio* species often remains unproven; therefore, they are usually regarded as opportunistic pathogens (Garnier et al., 2007; Jenkins et al., 2013; Go et al., 2017; De Lorgeril et al., 2018). Culture-dependent studies have observed shifts in the *Vibrio* community preceding the onset of disease in oysters, whereby putatively pathogenic *Vibrio* bacteria replaced the benign *Vibrio* commensals (Lemire et al., 2015). To better understand the role of *Vibrio* species in disease events such as those afflicting oysters, a precise high throughput technique that characterizes this important bacterial group is required.

Here, a new *Vibrio*-centric *hsp60* amplicon sequencing assay was developed, coupled with a customized *Vibrio hsp60* sequence reference database, to precisely characterize *Vibrio* diversity in environmental samples using the Illumina sequencing platform. The utility of this new assay was first validated using mock *Vibrio* communities constructed from varying proportions of *Vibrio* isolates before moving to tests with natural seawater samples, and finally applying it to characterize patterns in *Vibrio* diversity during a Pacific oyster mortality event (Green et al., 2019).

## Materials and Methods

### Primer and *Vibrio* Reference Dataset Construction

In order to develop a reference dataset to aid the design of a new set of degenerate primers targeting the *hsp60* gene, 100 *Vibrio hsp60* coding sequences were collected from the NCBI repository and blasted against the NCBI nucleotide database (nt file). Sequences were extracted using extract_hitseqs_from_sequences.pl (Kahlke, 2019) and both accession numbers and their respective taxonomy were then extracted using list_basta_taxa.py provided by BASTA v1.3.2.3 (Kahlke and Ralph, 2019). The BLAST output was filtered to retain taxa assigned to the *Vibrio* genus using the filter_basta_fasta.py script also provided by BASTA (Kahlke and Ralph, 2019) and genes assigned as *hsp60* and *groEL* were collected and added to our *Vibrio*-*hsp60* dataset. Both of these genes were chosen because they have previously been assigned as the same gene, but are annotated differently (Silvester et al., 2017). The aforementioned steps generated 1926 *Vibrio hsp60*/*groEL* sequences. The *Vibrio*-*hsp60* dataset was then aligned with MAFFT (Katoh et al., 2017) using the einse–reorder option and the dataset was cleaned (removal of short sequences and those sequences with “N” base pairs), yielding 1468 *Vibrio hsp60*/*groEL* sequences across more than 30 different *Vibrio* species. The aligned untrimmed *hsp60* dataset was visualized using UGENE (Golosova et al., 2014) and highly conserved areas within the consensus sequence were chosen for primer construction. Primers were constructed using the Primer3Plus (Untergasser et al., 2007) software. The constructed degenerate primers were named Vib-hspF3-23 and Vib-hspR401-422 (Table 1), and their application resulted in the amplification of a 487 bp PCR product (Illumina adapters inclusive).

**TABLE 1:** Primers used in this study.

**Primer name**	**Sequence (5′–3′)**	**Source**
Vib-hspF3-23	TCGTCGGCAGCGTC AGATGTGTATAAGAGACAG GAACCCNATGGAYC TKAARCG	This study
Vib-hspR401-422	GTCTCGTGGGCTCGG AGATGTGTATAAGAGACAG GCVATGATHARHA GHGRRCGNG	This study
16S 341F	CCTACGGGN GGCWGCAG	Herlemann et al., 2011
16S 805R	GACTACHVG GGTATCTAATCC	Herlemann et al., 2011
VF169	TCGTCGGCAGCGTCAGA TGTGTATAAGAGACAG GGATAACYATTG GAAACGATG	Yong et al., 2006
Vib-680R	GTCTCGTGGGCTCG GAGATGTGTATAAGAG ACAGGAAATTCTACCC CCCTCTACAG	Thompson et al., 2004
Vib-567F	GGCGTAAAG CGCATGCAGGT	Thompson et al., 2004
Vib2-r^∗^	GAAATTCTACCC CCCTCTACAG	Thompson et al., 2004

*Underlined sequences are Illumina sequencing adapters. ^∗^Vib2-r is Vib-680R without the Illumina adapters included.*

To ensure that the *Vibrio* reference dataset was constructed with accurately assigned *Vibrio* taxa and not partial *hsp60* reads (which could possibly be assigned to the wrong taxa), we constructed a reference dataset using *hsp60* sequences taken from whole genomes. First, all of the currently available complete *Vibrio* genomes were collected from the NCBI repository (185 genomes) and a BLAST database was constructed using these genomes. *Vibrio hsp60* sequences were compared against this database and all hits at least 65% similar to the query *hsp60* sequence and at least 400 base pairs long were extracted using extract_hitseqs_from_sequences.pl (Kahlke, 2019). BLAST hits were then visualized and trimmed to the primer locations in MEGA (version 7.0.26). To determine the coverage of *Vibrio* species in our *Vibrio* reference dataset, we compared the taxa in this trimmed dataset against the listed *Vibrio* species in the NCBI taxonomy database. Where possible, *hsp60* sequences for missing *Vibrio* species were collected from incomplete whole genomes and added to the *Vibrio* reference dataset. This yielded a dataset comprising of 106 different *Vibrio* species incorporating 284 *hsp60* sequences. In some instances, *hsp60* was found in both *Vibrio* chromosomes. Where known, the second copy of the gene was named “group2.”

### Mock *Vibrio* Community Preparation

Ten *Vibrio* species, spanning five clades (Sawabe et al., 2013; Turner et al., 2018) and incorporating species that are relevant to both human health (Daniels and Shafaie, 2000) and aquaculture diseases (Luna-González et al., 2002; Bruto et al., 2017; Go et al., 2017) were grown overnight in LB20 broth (per liter: 10 g tryptone, 5 g yeast extract, 20 g NaCl), with shaking at 28°C. Bacterial cells were enumerated using a Beckman CytoFLEX flow cytometer and cell counts were diluted to a standardized concentration across all strains. Three different mock *Vibrio* communities were prepared by mixing the 10 *Vibrio* species in different dilution ratios (Table 2). DNA was then extracted from mock assemblages using the Qiagen DNeasy UltraClean Microbial Kit (catalog: 12224-250) following the manufacturer’s instructions.

**TABLE 2:** Composition of mock *Vibrio* communities generated in this study.

***Vibrio* species**	**Mock1 (%)**	**Mock2 (%)**	**Mock3 (%)**
*V. alginolyticus*	10	5	7.5
*V. campbellii*	10	5	7.5
*V. cholerae*	10	30	7.5
*V. crassostreae*	10	5	7.5
*V. diabolicus*	10	5	7.5
*V. harveyi*	10	5	20
*V. parahaemolyticus*	10	5	20
*V. rotiferianus*	10	5	7.5
*V. sinaloensis*	10	5	7.5
*V. vulnificus*	10	30	7.5

### Mock Community PCR Conditions and Sequencing

DNA extracted from the mock bacterial communities was diluted to 10 ng μL^–1^ and used in a 50 μL PCR reaction volume as follows: 10 μL of 5× Hi-Fi Buffer (Bioline), 5 μL of 10 mM dNTPs, 2 μL of high-fidelity velocity polymerase (0.5 units μL^–1^; Bioline), 2.5 μL of 10 μM forward primer (VF169 or Vib-hspF3-23), 2.5 μL of 10 μM reverse primer (Vib-680R or Vib-hspR401-422), 2 μL of DNA template (10 ng μL^–1^), with the remaining volume made up with sterile water. The PCR mixture was then subjected to the following PCR conditions: one cycle of 98°C for 2 min, 30 cycles of 98°C for 30 s, 50°C for 30 s, and 72°C for 30 s, and a final extension time of 72°C for 10 min. PCR products were purified with a Bioline Isolate II PCR and Gel Kit (catalog: BIO-52059) using the manufacturer’s instructions. For 16S (341F and 805R) rRNA sequencing, extracted DNA was amplified by the Ramaciotti Centre for Genomics with the following PCR conditions: 95°C for 3 min, 25 cycles of 95°C for 30 s, 55°C for 30 s, and 72°C for 30 s, and a final extension at 72°C for 5 min.

Mock bacterial community amplicons were characterized on the Illumina MiSeq platform (Ramaciotti Centre for Genomics; Sydney, NSW, Australia) using the manufacturers guidelines, using three primer sets (Table 1): the universal 16S rRNA primers 341F and 805R (Herlemann et al., 2011); a previously published *Vibrio*-specific 16S rRNA primer pair, VF169 (Yong et al., 2006) and Vib-680R (Thompson et al., 2004; Siboni et al., 2016); and the *Vibrio*-centric *hsp60* primer pair designed in this study, Vib-hspF3-23 and Vib-hspR401-422.

### Mock Community Sequence Analysis

Bacterial 16S rRNA and *hsp60* sequencing reads for the mock communities were processed as outlined in Kahlke (2018). Briefly, paired-end DNA sequences were joined using FLASH (Magoč and Salzberg, 2011) and subsequently trimmed using Mothur (Schloss et al., 2009) (PARAMETERS: universal 16S—maxhomop = 6, maxambig = 0, qaverage = 25, minlength = 491, maxlength = 501; *Vibrio*-specific 16S—maxhomop = 6, maxambig = 0, qaverage = 25, minlength = 533, maxlength = 534; *hsp60*—maxhomop = 6, maxambig = 0, qaverage = 25, minlength = 420, maxlength = 420). Trimmed sequence length was determined by the 2.5 and 97.5% tiles for the universal 16S and *Vibrio*-specific 16S assay, and 420 bp was chosen for the *hsp60* assay as it closely corresponded to the amplicon size (excluding illumina adapters) and was the 75% tile. The resulting fragments were clustered at 97% into operational taxonomic units (OTUs) and chimeric sequences were identified and removed using vsearch (Rognes et al., 2016). To assign taxonomy, QIIME (Caporaso et al., 2010) was used with the RDP classifier against either the Silva v128 database (for 16S rRNA analyzed samples) or against our custom *Vibrio*-*hsp60* reference dataset. Sequences were then rarefied to the same depth to remove the effect of sampling effort upon analysis.

### Seawater Collection, 16S rRNA Sequencing, and Data Analysis

To test the newly designed *Vibrio*-centric *hsp60* sequencing assay on seawater, water was collected from Sydney Harbour (33.839S, 151.254E) in the Austral summer. Seawater was filtered in triplicate through 0.22 μm membranes, before the filters were immediately snap frozen in liquid nitrogen. Microbial DNA was subsequently extracted from filters using a Qiagen DNeasy PowerWater kit (catalog: 14900-100-NF) and sent to the Ramaciotti Centre for Genomics (University of New South Wales, Sydney, NSW, Australia) for 16S rRNA (341F and 805R) sequencing on the Illumina MiSeq platform.

Raw 16S rRNA demultiplexed paired-end DNA sequences were joined using Flash (Magoč and Salzberg, 2011) and trimmed with Mothur (Schloss et al., 2009) (PARAMETERS: maxhomop = 6, maxambig = 0, qaverage = 25, minlength = 441, maxlength = 466); 96% of the sequences were successfully joined with FLASH and the trim lengths corresponded to the 25 and 97.5% tiles identified by Mothur. Fragments were then clustered into OTUs at 97% and chimeric sequences removed using vsearch (Rognes et al., 2016). QIIME (Caporaso et al., 2010) and the RDP classifier were then used to assign taxonomy against the Silva v128 database. Sequences were then rarefied.

DNA from seawater was also amplified using the Vib-hspF3-23 and Vib-hspR401-422 primer pair with the Illumina adapters added to the primers (Table 1). The 30 μL PCR reaction mixture was as follows: 6 μL of 5× Hi-Fi Buffer (Bioline), 3 μL of 10 mM dNTPs, 0.5 μL of high-fidelity velocity polymerase (2 units μL^–1^; Bioline), 1.5 μL of 20 μM forward primer, 1.5 μL of 20 μM reverse primer, 3.5 μL of template DNA, and the remainder (14 μL) made up with sterile water. The mixture was used with the following touchdown PCR conditions: one cycle of 98°C for 2 min, 5 cycles of 98°C for 30 s, 60°C for 30 s, and 72°C for 45 s, 21 cycles of 98°C for 30 s, 60°C for 30 s with a reduction of 0.5°C per cycle (60 to 50°C), and 72°C for 45 s, 16 cycles of 98°C for 30 s, 50°C for 30 s, and 72°C for 45 s, and a final extension time of 72°C for 10 min. Amplicons were then purified by the Ramaciotti Centre for Genomics, and characterized on the Illumina MiSeq platform using the manufacturer’s guidelines.

As the *Vibrio*-centric *hsp60* primer pair in some scenarios was found to non-specifically amplify other (non-*Vibrio*) taxa, a further cleaning step was added to the data analysis. In the first instance, pair-ended sequences were joined using FLASH (Magoč and Salzberg, 2011) and trimmed using mothur (Schloss et al., 2009) (PARAMETERS: maxhomop = 5, maxambig = 0, qaverage = 25, minlength = 420, maxlength = 420). These fragments were then clustered at 97% into OTUs and chimeric sequences were removed using vsearch (Rognes et al., 2016). Non-*Vibrio* sequences were removed by BLASTing cleaned sequences against the *Vibrio*-*hsp60* reference dataset and retaining those that were 90% similar to any sequence in that dataset. This fasta file was then used to assign taxonomy against the custom *Vibrio*-*hsp60* reference dataset with the RDP classifier. Due to the large spread of sequences per sample, data were not rarefied, rather sequences were normalized to the number of sequences per sample to produce the relative abundance of each taxa for each sample (data analysis workflow is available at King et al., 2019b; https://doi.org/10.17605/OSF.IO/4798P).

### Quantitative PCR (qPCR)

To provide an indication of *Vibrio* abundance, a quantitative PCR (qPCR) assay was used to quantify the number of *Vibrio*-specific 16S rRNA gene copies in each sample using the *Vibrio*-specific 16S rRNA gene primers Vib-567F and Vib2-r (Table 1) (Thompson et al., 2004). qPCR was performed using an epMotion 5075l Automated Liquid Handling System on a Bio-Rad CFX384 Touch Real-Time PCR Detection System with a seven-point calibration curve and a negative control. The calibration curve was created from 10-fold dilutions of a known quantity of amplicon DNA, measured by a Qubit fluorometer. All sample analyses were performed with three technical replicates, using the following reaction mixture: 2.5 μL iTaq Universal SYBR Green supermix, 0.4 mM of each forward and reverse primer, 1 μL of template DNA (50 ng μL-^1^), and the remainder made up with water. The qPCR cycling conditions were previously described (Siboni et al., 2016) and as follows: 95°C for 3 min followed by 45 cycles of 95°C for 15 s and 60°C for 1 min. The resulting data were normalized to milliliters of collected water. A coefficient of variation (CV) was then calculated for the technical triplicates, and where necessary, samples with CV > 1% had a replicate removed from the analysis. A melting curve was added to the end of every run to confirm the presence of a single PCR product.

### Laboratory-Induced Oyster Mortality Event

The newly designed *Vibrio*-centric *hsp60* primer set was applied to examine patterns in *Vibrio* community diversity during a previously described laboratory-induced Pacific Oyster mortality event, where *Vibrio* species had previously been implicated as the cause of oyster mortality during a simulated marine heatwave (Green et al., 2019). Briefly, triploid Pacific oyster (*Crassostrea gigas*) spat were collected from Port Stephens, New South Wales, Australia, prior to a forecasted marine heat wave. Spat were held in two different temperature conditions (low 20 ± 1°C and high 25 ± 1°C) with and without antibiotics (100 units/mL of penicillin and 0.1 mg/mL of streptomycin) and monitored for 6 days. Spat were placed in sterilized glass tanks, and UV and 5 μm filter sterilized seawater were added and replaced daily. Triplicate oyster spat were sampled on days 0, 3, 4, 5, and 6, and dead oysters were removed and frozen at -80°C prior to processing. Cumulative mortality was 77.4 ± 10.7 and 3.4 ± 5.9% for the high and low temperature treatment, respectively, with antibiotics in the high temperature treatment reducing the cumulative mortality to 4.3 ± 3.7%, indicating a likely role of bacteria in the oyster mortality. Mortalities were greatest between days 3 and 5. For the purposes of this study, DNA extracted from spat exposed to the high and low temperature treatments from each sampling point were amplified with the *Vibrio*-centric *hsp60* primer pair (Table 1). *Vibrio* dynamics were previously characterized within oyster tissues using a combination of culture-based approaches, qPCR and 16S rRNA amplicon sequencing (Green et al., 2019). Oyster DNA were subject to *hsp60* PCR amplification, sequencing, and data analysis, and qPCR, as described above for the seawater samples, with the exception of DNA being diluted to 50 ng μL^–1^ for the PCR conditions.

### Statistical Analyses

Comparisons of community compositions were performed using non-metric multidimensional scaling analysis (nMDS) with a Bray–Curtis dissimilarity index, applied to data normalized to the number of sequences per sample, with sequences less than 1% relative abundance removed, and then transformed (square root). Patterns observed in the nMDS analysis were statistically tested with a one-way PERMANOVA with 9999 permutations. To examine the similarity between each characterized community to the mock community, similarity indices were calculated with a Bray–Curtis dissimilarity index using data that were filtered, transformed, and with sequences assigned to the second chromosome (group 2) combined with their respective assigned species. To examine the contribution of individual *Vibrio* species to community dissimilarity, a SIMPER analysis with untransformed data was used with a Bray–Curtis dissimilarity index. To determine the relationship between *Vibrio*-specific 16S rRNA gene copies and the yield of cleaned *hsp60* sequences from the QIIME analysis, an ordinary least squares linear regression was used. Spearman’s rank correlation was used to examine relationships between *Vibrio* species (summarized at the species level) and oyster mortality. All statistical comparisons were performed in the PAST statistical environment (Hammer et al., 2001).

## Results and Discussion

### Comparison of *Vibrio* Mock Community Characterization Using 16S rRNA and *hsp60*

**FIGURE 1:**
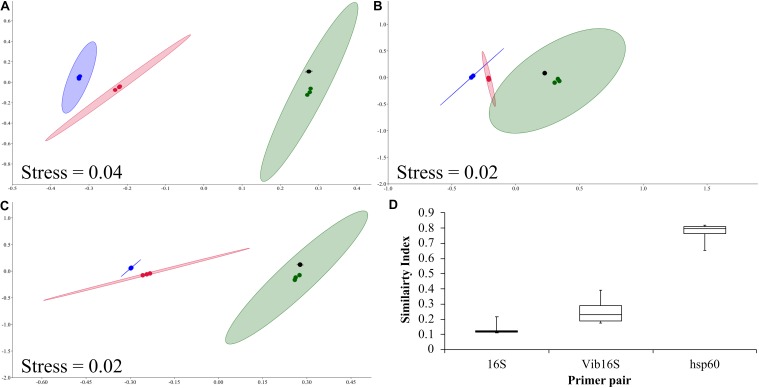
nMDS analysis of the 16S rRNA (blue dots), *Vibrio*-specific 16S rRNA (red dots), and *Vibrio*-centric *hsp60* (green dots) characterized mock communities, and the true mock community (black dots). Mock communities 1, 2 and 3 are **(A)**, **(B)** and **(C)** respectively. 95% ellipses are shown. **(D)** Box and whisker plot of Bray–Curtis similarity comparisons of community composition compared to the true mock communities. Data for all three mock communities are combined. For species assigned across two taxonomic assignments (e.g., group 2), they were combined with their respective species for **D**.

Mock *Vibrio* communities consisting of 10 different *Vibrio* species were characterized using the 16S rRNA, *Vibrio*-specific 16S rRNA, and *Vibrio*-centric *hsp60* primer pairs followed by Illumina MiSeq sequencing of the amplicons. When examined on an nMDS, the *Vibrio* community structure defined by the *Vibrio*-centric *hsp60* assay clustered closer to the true mock community than the two 16S rRNA-bases assays, with the true mock community sitting within the 95% ellipses for each *Vibrio*-centric *hsp60* characterized community (Figures 1A–C). Comparatively, the compositions of the 16S rRNA, *Vibrio*-specific 16S rRNA, and *Vibrio*-centric *hsp60* characterized communities were on average 16, 25, and 77% similar to the true mock community, respectively (Figure 1D).

**FIGURE 2:**
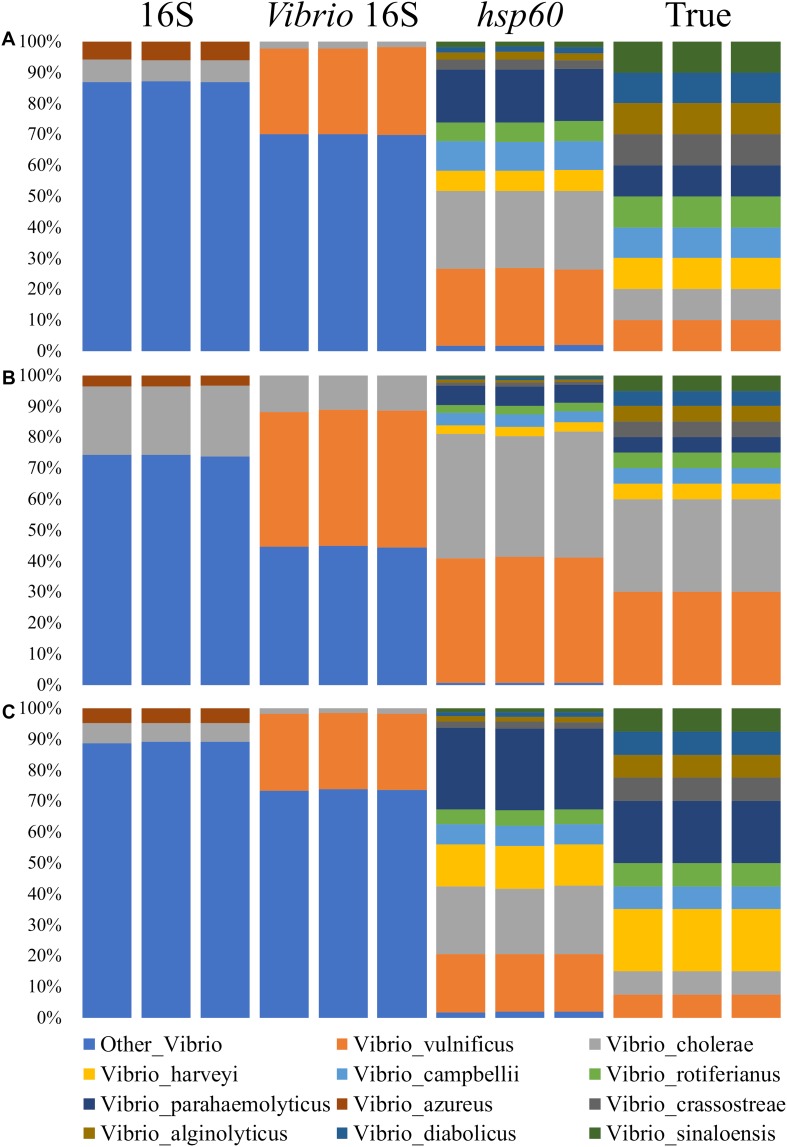
Comparison of amplicon sequenced phylogenetic markers for the *Vibrio* mock communities. Mock communities 1, 2 and 3 are **(A)**, **(B)** and **(C)** respectively. Each bar represents the average across a biological triplicate for each sequencing assay. Communities were characterized using 16S rRNA V3-V4 (Herlemann et al., 2011), *Vibrio*-specific 16S rRNA (Thompson et al., 2004; Yong et al., 2006), and *Vibrio*-centric *hsp60* primer pairs. The true mock community composition is also shown. Displayed data are relative abundance summarized at the species level. For sequences assigned to the second chromosome (group 2), they were combined with their respective species. Sequences representing less than 1% of the relative abundance were removed.

The *Vibrio* community data derived from the traditional V3–V4 16S rRNA primer pair was poorly characterized beyond the genus level, with only one *Vibrio* species in the mock community correctly identified (Figure 2). On average, sequences were not defined beyond the *Vibrio* genus level 90, 74, and 89% of the time in mock communities 1, 2, and 3, respectively. Of the sequences that were assigned beyond the *Vibrio* genus level, they were only assigned to *Vibrio cholerae* and *Vibrio azureus*. Notably, *V. azureus* was not part of the mock communities indicating not only imprecise, but incorrect taxonomic classification. *V. azureus* is within the *Vibrio harveyi* clade, for which it was probably incorrectly attributed (Yoshizawa et al., 2009). Sequences assigned to *V. cholerae* were correctly assigned but were marginally under-represented when compared to the real abundance within the mock community (6–23% for 16S rRNA; 7.5–30% for the mock communities).

Similar to the 16S rRNA characterized community composition, the data derived from the *Vibrio*-specific 16S rRNA sequencing assay were only able to identify two *Vibrio* species in the mock community, with the majority of the sequences not resolved beyond the genus level. Although correctly identified as *Vibrio*, the majority of sequences could not be assigned to the species level 70, 45, and 74% of the time in mock communities 1, 2, and 3, respectively. For the remainder of the sequences assigned beyond the genus level, they were correctly assigned to *Vibrio vulnificus* and *V. cholerae*. Sequences assigned to *V. vulnificus* were over-represented when compared to the true mock community composition (25–44% for *Vibrio*-specific 16S rRNA; 7.5–30% for the mock communities), while sequences assigned to *V. cholerae* were under-represented (2–12% for *Vibrio*-specific 16S rRNA; 7.5–30% for the mock communities).

Relative to both of the 16S rRNA based sequencing assays, the *Vibrio*-centric *hsp60* primer set identified the greatest number of species in the *Vibrio* community, with all of the species present in the mock community correctly identified by this assay. While all of the species were correctly identified, differences in the relative abundance of each species were observed when compared to the true mock community (Table 3). *Vibrio campbellii* was the best represented species with each *Vibrio*-centric *hsp60* characterized mock community only showing a 1% difference to the true mock community, while *Vibrio sinaloensis* was the most under-represented species with differences of 4–8% for the *Vibrio*-centric *hsp60* characterized communities. For *V. vulnificus* and *V. cholerae*, the only two correctly identified species in the 16S rRNA and *Vibrio*-specific 16S rRNA characterized communities, the *Vibrio*-centric *hsp60* assay provided better representation for *V. vulnificus* (over-representation of 10–13% compared to the mock community) compared to the *Vibrio*-specific 16S rRNA assay (over-representation of 14–18%) and 16S rRNA assay (not identified). For *V. cholerae*, this species was under-represented in both the 16S rRNA (1–8%) and *Vibrio*-specific 16S rRNA assays (6–20%) compared to an over-representation in the *Vibrio*-centric *hsp60* assay (9–14%). The exaggeration of *V. vulnificus* and *V. cholerae* could possibly be due to a greater *hsp60* primer affinity to these species, or the presence of two copies of *hsp60* in the genomes of these bacteria (one copy was identified in each chromosome for these two species).

**TABLE 3:** Relative abundance comparisons between the *Vibrio*-centric *hsp60* characterized mock communities and the true mock communities.

**Taxa**	**hsp60_1**	**True_1**	**Difference_1**	**hsp60_2**	**True_2**	**Difference_2**	**hsp60_3**	**True_3**	**Difference_3**
*V. alginolyticus*	7.4	10	–2.6	2.7	5	–2.3	5.7	7.5	–1.8
*V. campbellii*	8.8	10	–1.2	3.8	5	–1.2	6.2	7.5	–1.3
*V. cholerae*	23.9	10	13.9	39.2	30	9.2	20.9	7.5	13.4
*V. crassostreae*	2.9	10	–7.1	1.1	5	–3.9	1.9	7.5	–5.6
*V. diabolicus*	1.8	10	–8.2	0.8	5	–4.2	1.4	7.5	–6.1
*V. harveyi*	6.4	10	–3.6	2.8	5	–2.2	13.1	20	–6.9
*V. parahaemolyticus*	16.2	10	6.2	6.1	5	1.1	25.3	20	5.3
*V. rotiferianus*	6	10	–4	2.6	5	–2.4	4.7	7.5	–2.8
*V. sinaloensis*	1.6	10	–8.4	0.6	5	–4.4	1.2	7.5	–6.3
*V. vulnificus*	23.4	10	13.4	39.8	30	9.8	17.9	7.5	10.4
Other_*Vibrio*	1.7	0	1.7	0.6	0	0.6	1.8	0	1.8

*Displayed 1% filtered relative abundance is averaged across three replicates and in those cases where reads were assigned to the second chromosome (group 2), they were combined with their respective species. Relative abundance differences are also shown. 1, 2, and 3 represent mock communities 1, 2, and 3, respectively.*

Notably, the *Vibrio*-centric *hsp60* sequencing assay also distinguished members of the *V. harveyi* clade, a tight phylogenetic group within the *Vibrio* genus (Sawabe et al., 2013; Urbanczyk et al., 2013), which has previously had numerous incorrect taxonomic assignments to species within this clade caused by close 16S rRNA genetic similarity (Lin et al., 2010; Sawabe et al., 2013; Urbanczyk et al., 2013). This clade includes *Vibrio parahaemolyticus*, *Vibrio alginolyticus*, *V. harveyi*, *V. campbellii*, *Vibrio diabolicus*, and *Vibrio rotiferianus* (Sawabe et al., 2013; Turner et al., 2018), all of which were discriminated with the *Vibrio*-centric *hsp60* sequencing assay. Many of these species are important pathogens (Daniels and Shafaie, 2000; Luna-González et al., 2002; Go et al., 2017) and therefore accurately identifying their presence in environmental samples is an important requisite of a *Vibrio* specific assay of this type.

Previous attempts to perform *hsp60* amplicon sequencing have used universal *hsp60* primers and filtered the data for the assigned *Vibrio* sequences (Jesser and Noble, 2018). However, only 0.5% of the total *hsp60* data were assigned to *Vibrio* species in this previous study, and of these a significant proportion was unassigned to *Vibrio* species (Jesser and Noble, 2018), which was attributed to poor *Vibrio* species representation in the *cpn60* database (Hill et al., 2004; Jesser and Noble, 2018). In contrast, our assay resulted in 21.1% of the data being retained, with 98.1–99.5% of this being successfully assigned at the species level. We believe that the higher specificity of our assay was provided in part by the boutique *Vibrio hsp60* reference dataset that we developed, which encompasses 106 different *Vibrio* species compared to only 63 unique species in the *cpn60* database (accessed August 2019).

### *Vibrio* Diversity in Seawater

**FIGURE 3:**
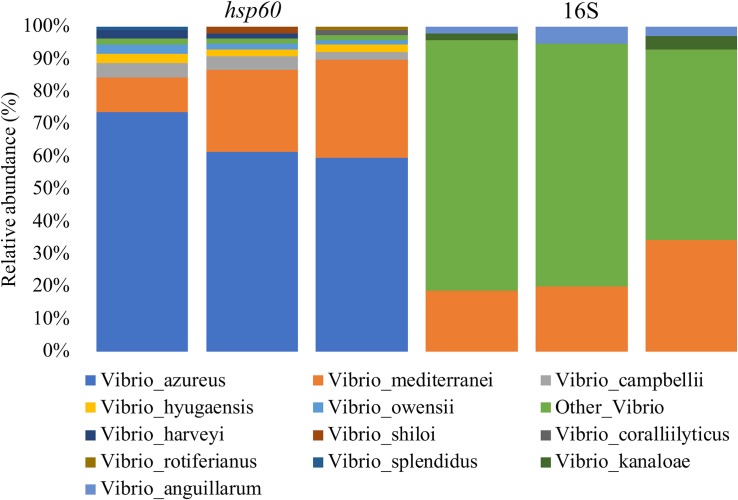
*Vibrio* diversity in seawater from Sydney Harbour. DNA was characterized with the *Vibrio*-centric *hsp60* and 16S rRNA V3-V4 (Watermann et al., 2008) primer sets. Displayed data are relative abundance summarized at the species level.

After confirming the utility of the *Vibrio*-centric *hsp60* sequencing assay using mock communities, this assay was used to characterize *Vibrio* diversity in seawater samples collected from Sydney Harbour, with the measured community composition compared to that derived from traditional 16S rRNA sequencing (Figure 3). Sequences assigned to the *Vibrio* genus or *Vibrio* species only made up 0.13–0.17% of the total bacterial community using 16S rRNA sequencing, with the majority (59–77% relative abundance) of these sequences not resolved beyond the *Vibrio* genus level. In contrast, only 1.4–1.7% of the sequences when using the *Vibrio*-centric *hsp60* sequencing assay were not assigned to a *Vibrio* species. Ten different *Vibrio* species were identified within the seawater samples using the *Vibrio*-centric *hsp60* sequencing assay, most of which occurred in low abundance (1–4% relative abundance) except for *V. azureus* and *Vibrio mediterranei*, which comprised between 58–71 and 10–29% of the total *Vibrio* community, respectively. In contrast, only three *Vibrio* species were identified using the 16S rRNA assay. Both assays identified the presence *of V. mediterranei*, with similar levels of relative abundance (16S rRNA: 19–34%; *hsp60*: 10–29%). The capability of the *Vibrio*-centric *hsp60* sequencing assay to resolve more sequences to the species level in seawater samples, relative to traditional 16S rRNA sequencing, highlights its utility when examining fine-scale patterns in *Vibrio* diversity.

### *Vibrio* Abundance Determines Assay Efficacy

**FIGURE 4:**
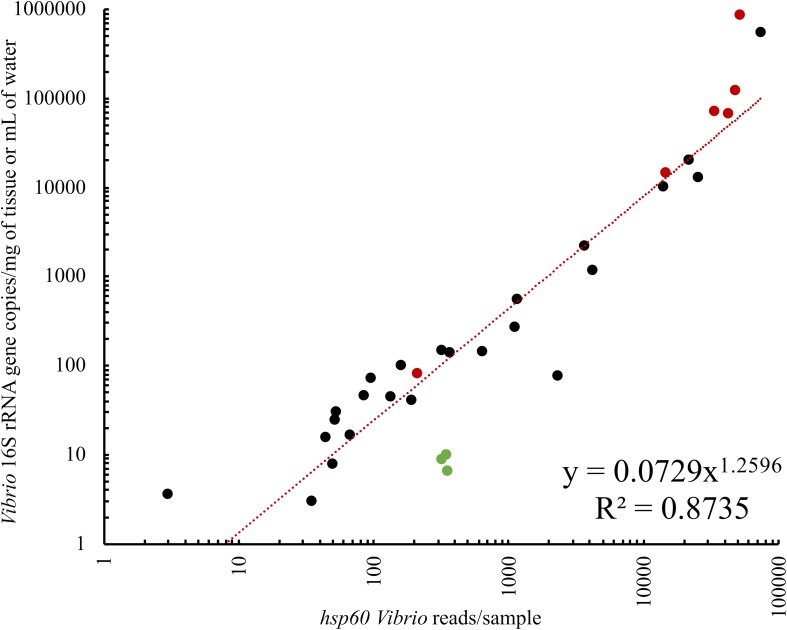
Ordinary least squares linear regression of *Vibrio* 16S rRNA gene copies and *hsp60* sequences per sample. *Vibrio* 16S rRNA gene copies were determined with qPCR while *hsp60* sequences refer to the analyzed Illumina sequencing data generated from the *Vibrio*-centric *hsp60* assay. Black dots are oyster samples, red dots are oyster mortality samples, and green dots are seawater samples. Both axes are logarithmic in scale.

To determine whether *Vibrio* abundance influenced assay data yield, both seawater and oyster samples were used. As expected, samples with the greatest abundance of *Vibrio*, as determined using qPCR targeting *Vibrio* 16S rRNA gene copies, had the greatest number of *hsp60* sequences (Supplementary Figure S1), with a significant relationship observed between *Vibrio* 16S rRNA gene copies and *hsp60* sequences (*R*^2^ = 0.87; *p* = 0.0001) (Figure 4). It is possible that the low number of *hsp60* sequences in samples with low *Vibrio* biomass was due to non-specific amplification of *hsp60* sequences associated with other bacterial genera. However, the linear relationship between *Vibrio* abundance and *hsp60* data yield indicates that when elevated levels of *Vibrio* are present within a sample, this assay delivers substantial capacity to probe the diversity of the community.

### *Vibrio* Diversity During a Laboratory Induced Oyster Mortality Event

After confirming the utility of the *Vibrio*-centric *hsp60* assay to track *Vibrio* community dynamics with high fidelity using a mock community and successfully applying it to characterize *Vibrio* diversity within natural seawater samples, it was next used to examine patterns in *Vibrio* diversity during a induced oyster mortality event. During a simulated heatwave event described in detail in Green et al. (2019), significant levels of oyster mortality were observed in oysters exposed to an increase in water temperature to 25°C (77.4 ± 10.7%), relative to oysters maintained at ambient temperature levels at 20°C (3.4 ± 5.9%). The *Vibrio*-centric *hsp60* assay was applied on samples derived from this study, because previous analyses (culturing and 16S rRNA sequencing) indicated a potential involvement of *Vibrio* in the oyster mortality event (Green et al., 2019).

**FIGURE 5:**
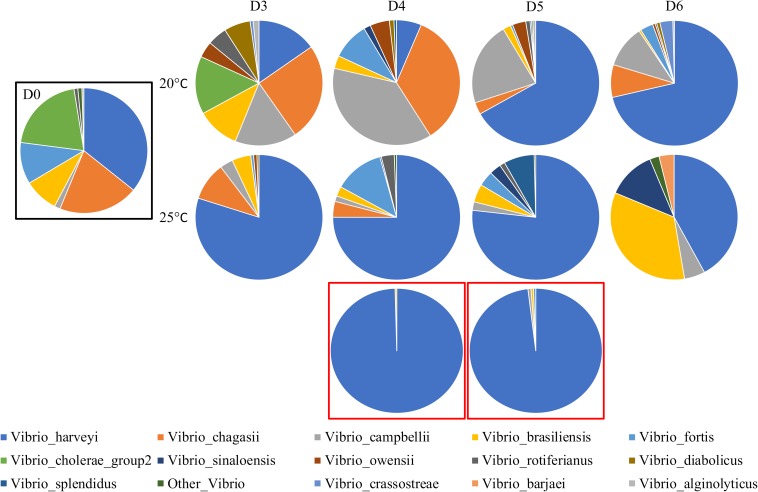
*Vibrio* community of *C. gigas* spat across 6 days and two temperature treatments. D0, D3, D4, D5, and D6 correspond to sampling days 0–6. Communities are averaged across three biological replicates and summarized at the species level. Communities in a black box are day 0. Communities in red boxes are dead *C. gigas* spat from the high (25°C) temperature treatment, taken on days 4 and 5, respectively. Sequences representing less than 1% of the relative abundance were removed.

Using the *Vibrio*-centric *hsp60* sequencing assay, the *Vibrio* community composition associated with Pacific oysters was significantly different between temperature treatments (*F* = 6.5, *p* = 0.0005; Supplementary Figure S2) and oyster mortality levels (*F* = 14.8, *p* = 0.0003 versus low temperature; *F* = 4.4, *p* = 0.013 versus high temperature). The “baseline” *Vibrio* community (Figure 5) on the first day of the experiment (day 0), 4 days prior to significant mortalities, was distributed across nine different species, with *Vibrio brasiliensis*, *Vibrio chagasii*, *Vibrio fortis*, and *V. harveyi* representing the dominant members of the *Vibrio* community with average relative abundances of 9, 20, 11, and 35%, respectively.

When comparing the control and marine heatwave samples, the *Vibrio* communities were on average 56% dissimilar to each other. In the ambient temperature control, *V. campbellii* and *V. chagasii* were the most prominent members of the *Vibrio* community, contributing 18 and 15% to the community dissimilarity (21 and 17.6% average relative abundance, respectively; Supplementary Table S1), with the relative abundance of both species negatively correlated to temperature (*r*_*s*_ = -0.4, *p* = 0.04; *r*_*s*_ = -0.53, *p* = 0.008, respectively; Supplementary Table S2) and oyster mortality (*r*_*s*_ = -0.45, *p* = 0.02; *r*_*s*_ = -0.52, *p* = 0.007, respectively). On the other hand, *V. harveyi* dominated the *Vibrio* community in the high temperature marine heatwave treatments, whereby this species contributed 37% to the community dissimilarity between temperature treatments and was positively correlated to temperature (*r*_*s*_ = 0.52, *p* = 0.011) and oyster mortality (*r*_*s*_ = 0.55, *p* = 0.006). On days 3–5, *V. harveyi* increased substantially in relative abundance from 35% on day 0 to 73–75% of the whole community, followed by a decrease in relative abundance (41%) on day 6. This pattern is consistent with the results of a *V. harveyi* specific qPCR assay previously performed on these samples, where a significant increase in copies of the *V. harveyi* gyrase B gene was observed on days 3–5, followed by a decrease on day 6 (Green et al., 2019).

Notably, a sharp increase in the relative abundance of *V. harveyi* was also observed in the low temperature treatment on days 5 (6% on day 4 to 65%) and 6 (68%), which was again consistent with qPCR data (Green et al., 2019). Dead oyster samples collected on days 4 and 5 from the high temperature treatment were also completely dominated by *V. harveyi*, which represented 97 and 96% of the *Vibrio* community, respectively. Low levels of oyster mortality (2%) were observed in the low temperature treatment on day 6 (Green et al., 2019), which notably corresponded with an increase in the relative abundance of *V. harveyi* on the preceding day (6–65% from days 4 to 5), possibly indicating a time-delayed relationship for *V. harveyi* proliferation due to the lower temperature. *V. harveyi* was previously implicated as the causative agent behind this mortality event (Green et al., 2019) and a previous study implicated *V*. *harveyi* as a causative agent for an unknown mass mortality outbreak from the same region the oysters were sourced from (Port Stephens) (Go et al., 2017). The data derived from the *Vibrio*-centric *hsp60* sequencing approach were able to unambiguously pinpoint the putative pathogen that increased in abundance prior to disease onset, as evidenced by previous culturing studies (Go et al., 2017; Green et al., 2019).

During the simulated marine heatwave, temperature was strongly correlated with oyster mortality (*r*_*s*_ = 0.87, *p* = 0.0001) and may have provided a selective advantage for *V. harveyi* allowing for an increase in the relative abundance of this species, effectively replacing the putative commensal *Vibrio* species (Lemire et al., 2015) and/or temperature may have acted as an immunosuppressant in the oysters allowing for a shift in the *Vibrio* community preceding disease (Lokmer and Wegner, 2015). Interestingly, the oysters on day 6 in the high temperature treatment had a decreased number of sequences assigned to *V. harveyi* relative to the preceding days (75 to 45% from days 5 to 6). A possible explanation for this pattern is that a sub-population of surviving oysters exhibited higher tolerance to the elevated temperature conditions, avoided colonization by *V. harveyi* and survived.

These results indicate that temperature stressed Pacific oysters undergo a substantial shift in the composition of their *Vibrio* community, involving a dramatic increase in the relative abundance of *V. harveyi*, which precedes oyster mortality. Both the occurrence of elevated levels of the *V. harveyi* in oysters before and during mortality and the very high levels of *V. harveyi* in freshly deceased oysters further implicate this species in oyster mortality events, in agreement with previous studies (Saulnier et al., 2010; Segarra et al., 2010; Jenkins et al., 2013; Le Roux et al., 2016; Go et al., 2017).

### Advantages and Disadvantages of the *Vibrio*-Centric *hsp60* Assay

Due to the non-specific nature of our primer set, sometimes a significant proportion of *hsp60* sequences was assigned to non-*Vibrio* taxa, including species assigned to the *Labrenzia*, *Erythrobacter*, *Phaeobacter*, and *Pseudomonas* genera (Supplementary Table S3), and were therefore removed. Despite this, the *Vibrio*-centric *hsp60* assay delivered significantly enhanced species identification in all of the tested samples, relative to 16S rRNA, with community profiles displaying high levels of fidelity with the known composition of mock communities. Relative to 16S rRNA and previously published universal *hsp60* primers (Jesser and Noble, 2018), our *hsp60* assay also generated a greater yield of *Vibrio-*assigned data, with our *hsp60* assays full potential attained in combination with our boutique *Vibrio*-database. In some instances, samples with a low *Vibrio* abundance will likely generate low numbers of cleaned *hsp60* sequences. In this study, we removed sequences that were not at least 90% similar to any *Vibrio hsp60* sequence in our boutique *Vibrio* database, which was derived from 106 different *Vibrio* species. The 90% threshold was chosen as lower thresholds (i.e., 85 or 80%) were found to include other taxa closely related to *Vibrio* (i.e., *Photobacterium*). While the number of sequences not assigned to a *Vibrio* species in our samples was low, it may be possible that some of these sequences could be unidentified *Vibrio* species or *Vibrio* species not in our *Vibrio* database. To determine the identity of these sequences assigned to the “*Vibrio*” genus, the representative sequence can be BLASTed against the NCBI database and where necessary, new *hsp60* sequences can be added to the *Vibrio* database.

## Conclusion

Most standard approaches for examining *Vibrio* diversity are constrained by poor taxonomic resolution beyond the genus level. This is often a significant limitation because *Vibrio* species are often implicated in disease events among both natural populations of marine organisms (Kushmaro et al., 2001; Austin and Zhang, 2006; Rubio-Portillo et al., 2014) and commercially important aquaculture species (Goarant et al., 2000; Becker et al., 2004; Frans et al., 2011; Geng et al., 2014; Vezzulli et al., 2015). Here, a *Vibrio*-centric *hsp60* sequencing assay was created using primers tailored to *Vibrio-*centric *hsp60* and used in combination with a custom-built *Vibrio* reference dataset including 106 *Vibrio* species. The sequencing assay was able to successfully identify every *Vibrio* species included within a mock community constructed with known dilutions of different *Vibrio* species. Despite an exaggeration in the relative abundance of some species, the *Vibrio*-centric *hsp60* sequencing assay provided greatly superior taxonomic resolution when compared to conventional 16S rRNA sequencing methods. The *Vibrio*-centric *hsp60* sequencing assay was subsequently successfully applied to seawater samples providing better discrimination of *Vibrio* diversity compared to 16S rRNA amplicon sequencing approaches, highlighting its utility in seawater. Next, the sequencing assay was able to unambiguously identify the *Vibrio* species that increased in abundance during an oyster mortality event, pinpointing a putative pathogen involved in the deaths of oysters following a simulated marine heatwave. This *Vibrio*-centric *hsp60* sequencing assay offers the potential for high throughput characterization of *Vibrio* diversity while retaining a highly specific degree of taxonomic resolution in environmental samples, important for dissecting species level community dynamics and their relationship with the environment or disease.

## Data Availability Statement

The raw fastq files generated for this study are available under BioProject number PRJNA580513. Data analysis pipeline is available at https://doi.org/10.17605/OSF.IO/4798P.

## Author Contributions

WK and NS created the degenerate primers and optimized the PCR. WK, NS, and TK analyzed the data. TG performed the oyster mortality experiment and provided samples. ML and JS conceived and designed the study. WK, NS, ML, and JS wrote the manuscript.

## Conflict of Interest

The authors declare that the research was conducted in the absence of any commercial or financial relationships that could be construed as a potential conflict of interest.
