# Supplementary material for: A New High Throughput Sequencing Assay for Characterizing the Diversity of Natural Vibrio Communities and Its Application to a Pacific Oyster Mortality Event
Source: Front Microbiol. 2019 Dec 20;10:2907. doi: 10.3389/fmicb.2019.02907 (PMC6932961; doi:10.3389/fmicb.2019.02907)
Supplement: Supplementary file 1 [file Data_Sheet_1.docx]

**Supplementary Information**

Supplementary Figure 1: Comparison of cleaned *hsp60* assigned sequences of each sample to *Vibrio*-specific 16S rRNA qPCR. Blue and red columns are samples from the low (20°C) and high (25°C) temperature treatment respectively, while green columns are water samples. The *hsp60* reads (primary x-axis; columns) were the highest in mortality samples (red hatched), followed by the high temperature treatment. The number of *Vibrio* 16S rRNA gene copies (secondary x-axis; black line) was elevated in accordance with the number of *hsp60* reads. Both axes are logarithmic in scale.

Supplementary Figure 2: non-metric multidimensional scaling analysis of oyster *Vibrio* community composition. Blue and red dots are the low and high temperature treatments respectively. Black dots are the first day of sampling (day zero) and purple dots are the dead oyster samples.

Supplementary Table 1: SIMPER analysis of *Vibrio* communities between temperature treatments. Dissimilarity contribution is provided with the average of each species in each treatment.

| *Vibrio* species | Dissimilarity contribution (%) | Low temperature average (%) | High temperature average (%) |
| --- | --- | --- | --- |
| *V. harveyi* | 37.07 | 38.8 | 72.5 |
| *V. campbellii* | 17.73 | 21 | 2.57 |
| *V. chagasii* | 14.91 | 17.6 | 4.12 |
| *V. brasiliensis* | 6.91 | 4.17 | 6.88 |
| *V. fortis* | 5.282 | 3.24 | 5.08 |

Supplementary Table 2: Spearman’s correlation between oyster mortality, temperature and *Vibrio* species. Data is presented as r_s_ value, p-value. NS is not significant.

| Variable | Mortality | Temperature |
| --- | --- | --- |
| *V. harveyi* | 0.55, 0.0057 | 0.52, 0.0011 |
| *V. chagasii* | -0.52, 0.0065 | -0.53, 0.008 |
| *V. campbellii* | -0.45, 0.025 | -0.41, 0.042 |
| *V. owensii* | -0.55, 0.0044 | -0.51, 0.014 |
| *V. sinaloensis* | NS | 0.41, 0.046 |
| Temperature | 0.87, 0.0001 | N/A |

Supplementary Table 3: Taxonomic classification of non-*Vibrio* reads that were removed during the standard data analysis workflow. Provided is the taxa name and the number of OTUs assigned to that taxa. Only those taxa with more than 2 assigned OTUs are shown.

| Taxa name | Number of OTUs |
| --- | --- |
| *Labrenzia* | 101 |
| *Erythrobacter flavus* | 45 |
| *Labrenzia sp. CP4* | 16 |
| *Labrenzia aggregata* | 10 |
| *Pseudomonas* | 10 |
| *Phaeobacter gallaeciensis* | 8 |
| *Erythrobacter seohaensis* | 8 |
| *Sphingorhabdus flavimaris* | 3 |
| *Pseudomonas fluorescens* | 3 |
| *Bacteria* | 2 |
| *Devosia sp. I507* | 2 |
| *Antarctobacter heliothermus* | 2 |
| *Celeribacter manganoxidans* | 2 |
| *Phaeobacter* | 2 |
| *Altererythrobacter namhicola* | 2 |
